# Reasons individuals stop eating questionnaire (RISE-Q) among adults in the United Arab Emirates

**DOI:** 10.1371/journal.pone.0293386

**Published:** 2023-10-25

**Authors:** Leila Cheikh Ismail, Tareq M. Osaili, Hanan Salem, Mona Abdelrahim, Nadin Gamaleldin, Noor Shalfawi, Razan Nasser, Tala Al Daour, Maysm N. Mohamad, Sheima T. Saleh, Rameez Al Daour, Haleama Al Sabbah, Abir Ajab, Lily Stojanovska, Ayesha Al Dhaheri

**Affiliations:** 1 Department of Clinical Nutrition and Dietetics, College of Health Sciences, University of Sharjah, Sharjah, United Arab Emirates; 2 Nuffield Department of Women’s & Reproductive Health, University of Oxford, Oxford, United Kingdom; 3 Department of Nutrition and Food Technology, Faculty of Agriculture, Jordan University of Science and Technology, Irbid, Jordan; 4 Department of Nutrition and Health, College of Medicine and Health Sciences, United Arab Emirates University, Al Ain, United Arab Emirates; 5 Public Health Department, College of Health Sciences, Abu Dhabi University, Abu Dhabi, United Arab Emirates; 6 Institute for Health and Sport, Victoria University, Melbourne, Australia; School of Health Binawan: Universitas Binawan, INDONESIA

## Abstract

The relationship between obesity and satiation is complex and bidirectional. Understanding differences in reasons for meal termination may enhance our understanding of overeating risks and susceptibility to overconsumption. This study aimed to investigate the reasons why individuals in the UAE stop eating. A cross-sectional web-based study was conducted among adults ≥18 years in the UAE (n = 1482). Using a self-administered online questionnaire, we collected information on sociodemographic characteristics, lifestyle habits, and eating behavior using the Reasons Individuals Stop Eating—Questionnaire (RISE-Q-15) used twice for breakfast/main meal. The items were categorized into five scales; decreased food appeal (DFA), physical satisfaction (PS), planned amount (PA), self-consciousness (SC), and decreased priority of eating (DPE). All items were scored from 1 to 7 ranging between 3 to 21 on each scale. A paired t-test was used to evaluate the difference between the RISE-Q scores on each scale concerning the two meals. The main reason why participants stopped eating breakfast was under the PS scale (14.91 ± 3.72), followed by the PA scale (14.58 ± 3.00). The main reason why participants stopped eating main meals was under the PS scale (14.78 ± 3.56), followed by the PA scale (14.77 ± 43.81). The mean score of the DPE scale was significantly higher for breakfast than the main meal (p = 0.038). More than half of the participants reported an average eating rate (58.7%). Pearson’s chi-square analysis revealed that the eating rate was dependent on BMI (p<0.001). Considering individual mealtimes and addressing factors related to PS and PA of food is crucial when designing nutrition interventions aiming to promote healthy eating habits among adults in the UAE.

## Introduction

Obesity is currently acknowledged as a disease of significant global concern, reaching epidemic levels. It can be defined as an excessive accumulation of body fat which can significantly increase risks of developing a range of chronic conditions, such as type 2 diabetes, cardiovascular disease, specific forms of cancer, and musculoskeletal disorders [1]. As per the World Health Organization (WHO), between 1975 and 2016, the prevalence of adults who are overweight or obese than tripled, reaching 39% and 13%, respectively [2]. In the United Arab Emirates (UAE), 67.9% adults aged 18–69 years were living with overweight in 2017–2018 and 27.8% were living with obesity [3].

A variety of factors have influenced eating habits in the UAE during the last 40 years such as rapid economic growth, urbanization, and nutrition transition from traditional foods to calorie-dense meals [4]. Energy intake is influenced by satiation; a key mechanism that regulates the termination of eating and controls the size of a meal [5]. Cunningham and Rolls have suggested a novel paradigm for satiation as a dynamic series of processes that leads to the conclusion of a meal [6] and developed the Reasons Individuals Stop Eating Questionnaire (RISE-Q) to investigate differences in reasons for ending a meal among individuals [7]. This new instrument characterizes individual reasons for meal termination which can enhance our understanding of the psychobiological processes involved in satiation and the causal mechanisms that contribute to overeating and potential weight gain. The tool originally contained 31 items but was then shortened into 15 items (RISE-Q-15) covering physical, psychological, environmental, and social domains [8]. Through exploratory factor analysis, researchers have identified five latent factors for meal termination: decreased food appeal (DFA), physical satisfaction (PS), planned amount (PA), self-consciousness (SC), and decreased priority of eating (DPE) [8].

Studies have shown that the relationship between obesity and satiation is complex and bidirectional, and eating cessation can be influenced by several behavioral and biological factors during the meal [9, 10]. For instance, in Finland, the main reason for stopping eating among adults was individual self-assessment of their feeling of satiety [11]. Moreover, a recent systematic review and meta-analysis revealed a relationship between poor interoceptive awareness including the weak ability to respond to satiety signals, and high body mass index (BMI) [12]. Therefore, understanding individual differences in reasons for meal termination in the context of interoceptive awareness could help to improve our understanding of the risk factors for overeating and to explain variability in energy intake and susceptibility to overconsumption [7]. Therefore, this study aimed to examine the reasons why people in the UAE stop eating during a meal and explore differences in the reason for meal termination between two major meals (breakfast and main meal) throughout the day. Additionally, it aimed to examine the association between BMI and participant self-reported eating rate (SRER).

## Methods

### Study design and participants

A population, web-based cross-sectional study was conducted between February and May of 2022 among adults from all seven emirates in the UAE. The following inclusion criteria were used to enroll participants in the study: (1) adults ≥ 18 years, (2) currently residing in the UAE, (3) without a history of eating disorders. This selection of the inclusion criteria aligns with the study objectives and ensures appropriate use of the RISE-Q which was previously validated on an adult population [8]. Participants were recruited using snowball convenience sampling techniques to achieve large-scale dissemination. The following calculation was used to obtain the minimal sample size required, with a 95% confidence interval:

$$N=z^{2}\times P\times (1-P)/e^{2}$$

Where z = 1.96; P = (estimated proportion of the population that presents the characteristic) = 0.5; e (margin of error) = 0.05; N (sample size) = 384 participants, plus 20% (attrition rate) = 460 participants. The questionnaire was developed using Google Document Forms. A uniform resource locator (URL) web link was generated and disseminated on social media platforms (e.g., LinkedIn^™^, Facebook^™^, and WhatsApp^™^) inviting participants to complete the questionnaire and encouraging them to share the link with their contacts. The online survey was completed by 1482 adults, which exceeded the anticipated sample size due to a high response rate, resulting in higher statistical power and enhanced external validity.

An information sheet explaining the study protocol and objective was provided on the first page. This also included screening questions to ensure that the participants were ≥18 years old, currently reside or live in the UAE, and do not have a history of eating disorders. Participants who read the information and provided electronic consent were able to proceed with answering some demographic questions, followed by lifestyle questions and the RISE-Q-15 questionnaire. The survey took approximately 10 minutes to be completed. Participants were allowed to exit the online survey at any point and were informed that their participation was anonymous and voluntary.

Ethical approval was obtained from the Research Ethics Committee at the University of Sharjah (UOS) (Reference number: REC_22-_02-_19–03_S). The study was conducted following the ethical standards laid down in the 1964 Helsinki Declaration. No identification information was collected to assure confidentiality and anonymity of participants. Electronic informed consent was obtained from all participants. Furthermore, the RISE-Q is copyrighted. Therefore, authorization from Professor Barbara Rolls was sought, and the questionnaire and scoring guide for RISE-Q and RISE-Q-15 were received.

### Study questionnaire

A self-administered multi-component online questionnaire was used in this study. The survey questionnaire included three sections; sociodemographic information, lifestyle habits, and the RISE-Q-15 survey. The socio-demographic section included questions on sex, age, nationality, Emirate of residence, education level, number of family members, academic level, self-reported weight (in kg) and height (in cm), and history of chronic disease.

The participant’s lifestyle section was adapted from previously published studies [13, 14] which inquired about smoking status, exercise frequency per week, frequency of doing household chores, screen time for work and entertainment, sleeping duration, daily water consumption, if participants are following any diet, if they consume breakfast daily, and the main meal they consume. A short food frequency questionnaire was also used [13]. Additionally, a question about Self Reporting Eating Rate (SRER) based on the study by van den Boer et al., was added which reflects participants’ actual eating rate (response options; Very slow, slow, average, fast, very fast) [15].

The last section included the RISE-Q-15 survey [8] which was used twice, once regarding a typical breakfast at home and once regarding a typical main meal (lunch or dinner) at home. The RISE-Q-15 survey has previously been validated by Chawner et al. [8]. A total of 15 statements asking participants how often each statement is a reason to stop eating were listed with answers rated on a seven-point frequency scale ranging from (1) Never to (7) Always. The statements were based on the identified scales for meal termination: (1) Decreased Food Appeal (DFA): “The food is no longer appealing to me”, “The food is no longer pleasant” and “I am no longer interested in the food”; (2) Physical Satisfaction (PS): “My stomach is full”, I am satisfied with the amount I have eaten”, and “The amount I have eaten matches my appetite”: (3) Planned Amount (PA): “I have eaten the amount that I planned”, “I have eaten the amount that I served myself”, and “I have eaten the same amount that I usually eat”; (4) Self-Consciousness (SC): “I feel embarrassed about the amount I have eaten”, “I have eaten more than others”, and “I want to eat the same amount as everyone else”; and (5) Decreased Priority of Eating (DPE): “Eating no longer feels worth the effort”, “I have become restless”, and “I am no longer thinking about food”. The statements were administered in a random order across participants and without identifying the scales.

The original RISE-Q-15 questionnaire was developed in English [8]; thus, for this research, it has been translated into the Arabic language by two certified independent translators and back-translated to English by two independent bilingual researchers following an internationally established methodology [16]. The questionnaire was subsequently reviewed by the research team and the final translation was approved by the authors and a panel of experts in the field. Content validity test for each item of the translated questionnaire was performed using the Waltz and Basel’ method by asking the expert panel to define each item as clear, relevant, and simple, using a four-part Likert scale [17]. The results indicated an Item-Level Content Validity Index (I-CVI) in the range of 0.96–1.0, with a Scale-Level Level Content Validity Index (S-CVI/Ave) of 0.99, suggesting that 99% of the items in the questionnaire were deemed to be clear, relevant, and simple by the panel. Internal consistency was assessed using Cronbach’s alpha (α) and suggested good overall reliability of the instrument with a Cronbach’s alpha = 0.86. Before launching the online survey, a pilot test was conducted on 30 participants to ensure clarity. Data from the pilot test was not included in the final analysis of the study.

### Statistical analysis

Descriptive analyses were used to summarize demographic and lifestyle characteristics, and these were recorded using frequencies and percentages for categorical data and mean (standard deviation, SD) for continuous variables, as appropriate. BMI as defined by the World Health Organization (WHO), was calculated by dividing weight in kilograms by the square of height in meters. Participants’ bodyweight status was categorized as underweight (BMI ≤ 18.5 kg/m^2^), normal weight (BMI 18.5 to < 25 kg/m^2^), overweight (BMI 25 to < 30 kg/m^2^), obese (BMI ≥ 30 kg/m^2^) [18].

During data analysis, the RISE-Q-15 statements were categorized into the following five scales (three items in each scale); decreased food appeal (DFA), physical satisfaction (PS), planned amount (PA), self-consciousness (SC), and decreased priority of eating (DPE). All items were scored from 1 to 7. Each answer choice of the seven was given a score according to the following: never = 1, rarely = 2, Seldom = 3, sometimes = 4, often = 5, usually = 6, always = 7. Then the mean score for answers in each scale was calculated by averaging the scores across the relevant items respectively and omitting any with missing responses. The higher the score in each category the more frequent experience of the reasons. The minimum score is 3 reflecting the low experience of the reasons, and the maximum score is 21 which reflects the highest relation to the reasons for eating cessation.

A paired t-test was used to evaluate whether the difference between the RISE-Q scores in each scale concerning the two meals was statistically significant. The paired t-test was performed on a sample size of 835 comparing the observations for breakfast and main meal excluding the missing data for those who do not consume breakfast (n = 648). The Self Reporting Eating Rate (SRER) was classified into three categories: slow, average, medium, and fast; the "very slow" category was combined with the "slow" category, and the "very fast" category was combined with the "fast" category for the analysis. A chi-square test was used to evaluate if the eating rate was dependent on BMI. *P* values at <0.05 were considered statistically significant. Data were analyzed using SPSS software, version 26.0 (SPSS, Chicago, IL, USA).

## Results

### Sociodemographic characteristics of the study participants

The total number of participants was 1482 of which 36.4% were males (Table 1), and the average age was 29.9 ± 11.2 years. Over, 25.3% were from Arabian Gulf countries, 40.3% from Levant countries (Lebanon, Jordan, Syria, Palestine), and 21.7% from North Africa. Over 23.9% were residing in Abu Dhabi, 37.2% in Dubai, and 27.1% in Sharjah. Over 5.8% of the participants were classified as underweight, 47.8% had normal weight, and 46.4% were overweight or obese. Most participants (69.4%) had a diploma/university education level and did not have a history of chronic diseases (87.7%).

**Table 1:** Sociodemographic characteristics of participants (n = 1482).

Variable	n (%)
Sex	
Females	942 (63.6)
Males	540 (36.4)
Nationality ^a^	
GCC countries	375 (25.3)
Levant	597 (40.3)
North Africa	322 (21.7)
Asia	117 (7.9)
Europe	10 (0.7)
Other	61 (4.0)
Emirate of residence	
Abu Dhabi	354 (23.9)
Dubai	552 (37.2)
Sharjah	402 (27.1)
Northern Emirates	174 (11.8)
BMI categories	
Underweight (<18.5 kg/cm^2^)	86 (5.8)
Normal weight (18.5–24.9 kg/cm^2^)	708 (47.8)
Overweight (25–29.9 kg/cm^2^)	432 (29.1)
Obese (≥ 30 kg/cm^2^)	256 (17.3)
Educational level	
High school or less	283 (19.1)
Diploma / University Education	1028 (69.4)
Higher education (Masters, Ph.D.)	171 (11.5)
History of chronic disease	
Yes	182 (12.3)
No	1300 (87.7)

Values represent frequencies and percentages [n, (%)]

^a^ (GCC: Gulf Cooperative Council, UAE, Bahrain, Kuwait, Oman, Qatar, Saudi Arabia). Levant area (Lebanon, Jordan, Syria, Palestine).

### Lifestyle characteristics of the study participants

The results showed that most of the participants (82.9%) do not smoke (Table 2). Over 41% stated never engaging in purposeful physical activity and 24.4% never do household chores. About one-third of the participants (36.7%) spend 5 hours/day on screen for work or study, while 18.8% reported spending more than 5 hours/day behind a screen for entertainment. Less than half of the participants consumed only 1–4 cups of water per day (41.7%), and only 19.9% met the recommended water consumption quantities. Over 16% of participants stated following a diet to lose weight. Only half of the participants (52.8%) met the recommended hours of sleep (7–9 hours) each night.

**Table 2:** Lifestyle characteristics of participants (n = 1482).

Variable	*n* (%)
Smoke	
Yes	254 (17.1)
No	1228 (82.9)
Exercise per week	
Never	620 (41.8)
1–2 times	579 (39.1)
3–5 times	229 (15.5)
More than 5 times	54 (3.6)
Doing household chores	
Never	362 (24.4)
1–3 times/week	601 (40.6)
4–5 times/week	141 (9.5)
Everyday	378 (25.5)
Screen time for study or work	
None	164 (11.1)
1–2 hour/day	322 (21.7)
3–5 hours/day	452 (30.5)
> 5 hours/day	544 (36.7)
Screen time for entertainment	
Less than 30 minutes/day	195 (13.2)
1–2 hours/day	544 (36.7)
3–5 hours/day	464 (31.3)
> 5 hours/day	279 (18.8)
Amount of water consumed per day	
1–4 cups	618 (41.7)
5–7 cups	569 (38.4)
8 cups or more	295 (19.9)
Type of current diet	
I am not following any diet plan	1175 (79.3)
To lose weight	243 (16.4)
To gain weight	33 (2.2)
For a medical reason	31 (2.1)
Breakfast consumption	
Yes	648 (43.7)
No	834 (56.3)
Main meal	
Lunch	1195 (80.6)
Dinner	287 (19.4)
Hours of sleep	
Less than 7 hours	625 (42.2)
7–9 hours	783 (52.8)
More than 9 hours	74 (5.0)

Values represent frequencies and percentages [n, (%)]

More than half of the participants did not consume fruits daily (57.7%) and 40.2% and 45.6% respectively did not consume vegetables and dairy daily (Table 3). Most participants (42.8%), reported the consumption of sweets and desserts 1–4 times/week, followed by 33.8% consumption once/day. Furthermore, over 17.4% of participants consumed sweetened drinks once/day. Most of the participants reported that they never consume energy drinks (71.9%).

**Table 3:** The frequency of consumption of foods among participants (n = 1482).

Food items	≥ 4 times/day	2–3 times/day	Once/day	1–4 times/week	Never
	*n* (%)				
Fruits	7 (2.9)	29 (12)	66 (27.4)	118 (49.0)	21 (8.7)
Vegetables	12 (5.0)	42 (17.4)	90 (37.3)	89 (36.9)	8 (3.3)
Milk and milk products	5 (2.1)	31 (12.9)	95 (39.4)	96 (39.8)	14 (5.8)
Meat/fish/chicken	3 (1.2)	22 (9.1)	120 (49.8)	92 (38.2)	4 (1.7)
Bread/rice/pasta	7 (2.9)	51 (21.2)	109 (45.2)	68 (28.2)	6 (2.5)
Sweets/desserts	2 (0.8)	28 (11.6)	71 (29.5)	117 (48.5)	23 (9.5)
Coffee/tea	20 (8.3)	66 (27.4)	74 (30.7)	65 (27.0)	16 (6.6)
Sweetened drinks (soda, juice)	1 (0.4)	13 (5.4)	42 (17.4)	124 (51.5)	61 (25.3)
Energy drinks	0 (0)	1 (0.4)	13 (5.4)	39 (16.2)	188 (78.0)

Values represent frequencies and percentages [n, (%)]

### Eating behavior

Participants were asked whether they consume breakfast meals regularly and those who did were further asked a set of questions to assess the reasons for stopping eating the breakfast meal. Similarly, participants were asked about their usual main meal (lunch or dinner) followed by an investigation of the reasons for stopping eating the main meal using the RISE-Q tool. The main reason why participants stopped eating their breakfast meal (n = 835) was under the physical satisfaction (PS) scale with a mean score of 14.91 ± 3.72, followed by the planned amount (PA) scale with a mean score of 14.58 ± 3.29 as shown in Table 4. Considering the mean scores of reasons to stop eating the main meal (n = 1482), the highest mean score was for the physical satisfaction (PS) scale with a mean score of 14.78 ± 3.56, followed by planned amount (PA) scale with a mean of 14.77 ± 3.81 (data available in S1 Table). Moreover, there was a statistical difference between the mean score of the Decreased Priority of Eating (DPE) scale between breakfast and the main meal, being significantly higher for breakfast compared to the main meal (p = 0.038).

**Table 4:** The difference in mean score for the five scales of the RISE-Q for breakfast and main meal among study participants (n = 835).

	Breakfast	Main Meal	p-value *
Scale	Mean ± SD	Mean ± SD	
Decreased Food Appeal	8.0 ± 3.9	8.1 ± 4.1	0.718
Physical Satisfaction	14.6 ± 3.3	14.4 ± 3.5	0.088
Planned Amount	14.9 ± 3.7	14.8 ± 3.8	0.293
Self-Consciousness	8.3 ± 3.6	8.3 ± 3.7	0.854
Decreased Priority of Eating	8.8 ± 3.4	8.6 ± 3.6	0.038

* Based on a paired sample t-test at a 5% level of significance.

**Fig 1:**
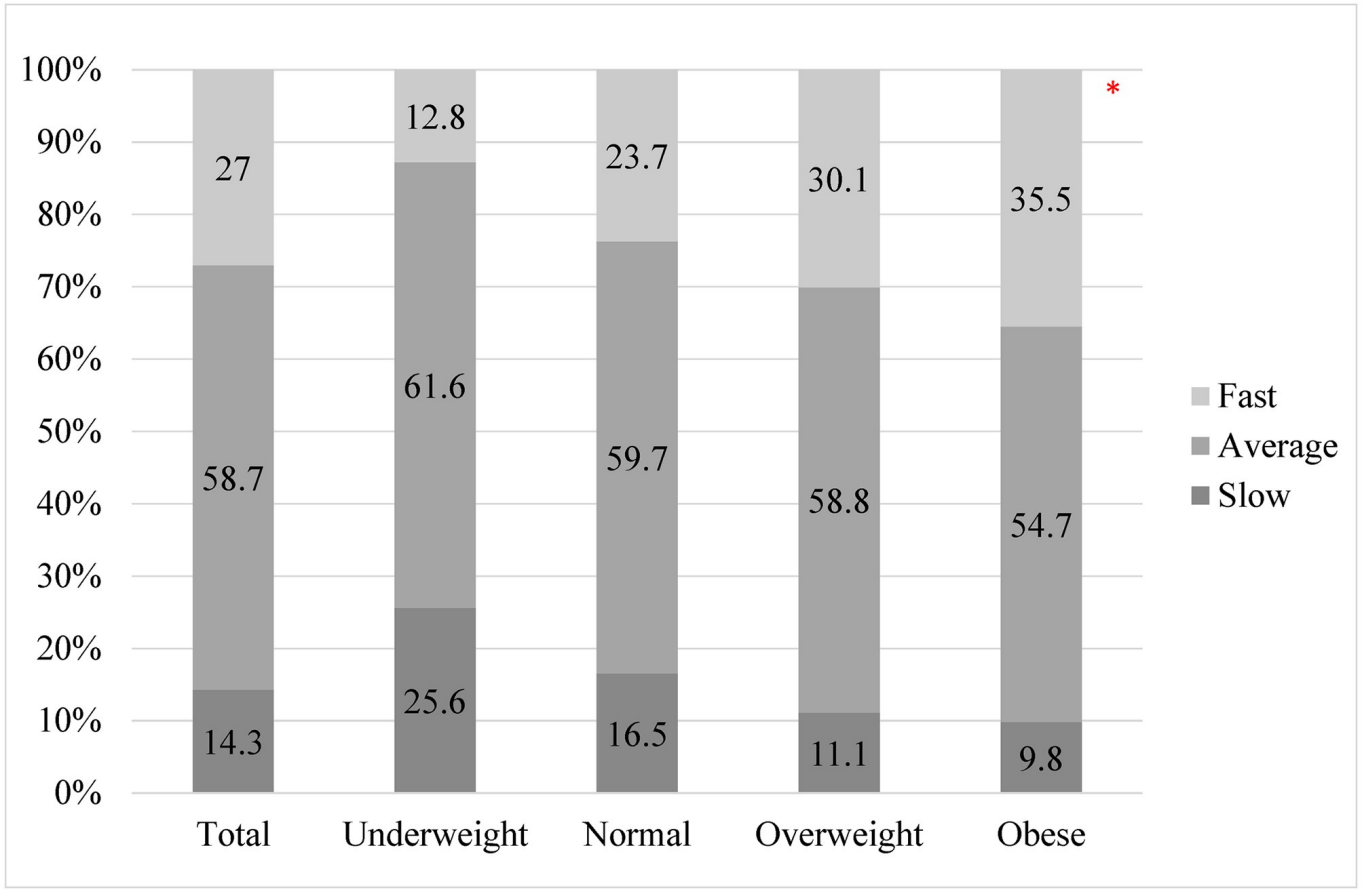
Self-reported eating rate based on BMI categories. * Indicates significance based on chi-square analysis.

Fig 1 illustrates the SRER among the participants based on BMI categories. Overall, most of the participants reported an average eating rate (58.7%), followed by fast (27.0%) and slow (14.3%). A chi-square test of independence was performed to evaluate the relationship between BMI categories and Eating rate. The relationship between these variables was significant (χ2 (2) = 35.513; p<0.001). Individuals classified as obese were more likely to report a fast-eating rate than normal weight and underweight participants.

## Discussion

The results of this cross-sectional study conducted among adults in the UAE highlight several important findings related to dietary habits and lifestyle factors that may impact food intake. Overall, the study found that the main RISE in the UAE was physical satisfaction and there was no significant difference between meals. Furthermore, the research findings highlighted that a substantial number of participants indicated insufficient physical activity, limited water intake, and subpar consumption of fruits, vegetables, and dairy products. Additionally, a notable portion of the participants reported elevated consumption of sugary treats, desserts, and sweetened beverages, all of which are recognized to carry adverse health implications when consumed excessively.

The results on lifestyle and eating behaviors were similar to a cross-sectional study conducted in the UAE during the COVID-19 lockdown which revealed that about half of the participants do not consume fruits daily and over one-third did not consume vegetables daily nor engage in physical activity [13]. Likewise, a study conducted during the lockdown in the UAE reported a high prevalence of unhealthy lifestyle behaviors among the UAE residents [19]. This effect remained unchanged after the availability of the COVID-19 vaccine [20] and it persists in the results of our current study.

The results indicated that the main reason for stopping eating was related to physical satisfaction and planned amount scales. This is consistent with previous research that showed individual self-assessment of their feeling of satiety as the main reason to stop eating among adults in Finland [11]. Similarly, a study aimed to explore the satiation framework and the processes that contributed to satiety using the RISE-Q, showed that the highest reported reason for stopping eating by individuals was physical satisfaction, followed by the planned amount [6]. Physical satisfaction in the RISE-Q was reflected by three statements including “the amount I have eaten matches my appetite” which highlights the importance of appetite in both initiation and cessation of eating, thus it is important to focus on appetite-related interventions when working with individuals living with obesity. Another study asked university students to complete the statement “I usually stop eating a meal when …” suggested that the most common response was “I feel full” which also reflects physical satisfaction [21]. Moreover, a crossover study investigated whether the total energy intake during the lunch meal influenced satiation and it showed that the predominant reason for stopping eating was fullness which also belongs to the physical satisfaction scale [22]. This emphasizes the importance of interoceptive awareness in regulating energy intake and controlling overconsumption. In general, interoceptive awareness refers to the ability to perceive and understand the signals originating from the body. It involves the recognition of internal cues such as heartbeat or breathing. In terms of food and eating, it refers to being aware of the internal cues for hunger and satiety [12, 23]. Therefore, interventions aimed at improving interoceptive awareness and self-monitoring of satiety signals could be useful in promoting healthy eating habits and reducing the risk of obesity.

The least scoring reason for stopping eating was decreased food appeal. Similarly, a study conducted on university students to report the reason for meal termination showed that the hedonic response of “the food tastes less pleasant” was the least selected response by only 8% of participants [21]. Likewise, a study conducted to explore the satiation processes reported that the least scoring reasons for stopping eating were decreased food appeal, followed by decreased priority of eating [6]. Decreased priority of eating refers to terminating the meal due to reduced motivation or a changed priority of eating [7, 24]. In the present study, a significant difference was observed in the mean score for the decreased priority of eating scale between breakfast and the main meal. One explanation could be that breakfast is the first meal of the day which is followed by different activities of the day, thereby reducing the priority of food compared to the main meal.

Regarding the self-reported eating rate, our results align with the van der Boer study [15], revealing a significant relationship between a high BMI and a fast eating rate. Participants with obesity reported a faster eating rate compared to both healthy individuals and those who were overweight. This was also similar to another study among adults indicating that faster eating rates are associated with higher adiposity as well as higher energy intake [25]. Evidence indicates that fast eating rates may be associated with increased risks of higher adiposity and developing metabolic syndrome [26]. In addition, a systematic review revealed that slower eating rates are associated with lower energy intake compared to faster eating rates [27]. This highlights the importance of looking into interventions to help people reduce their eating rate to aid in limiting excessive food consumption.

To our knowledge, this is the first study to assess the reasons for stopping eating different meals in the UAE. While this cross-sectional study provides important insights into the dietary habits and lifestyle factors that may impact food intake among adults in the UAE, there are several limitations to consider. Firstly, the study relies on self-reported data, which may be subject to recall bias or social desirability bias. Additionally, the study only included adults who were able to complete an online survey, which may not be representative of the broader population. Finally, the study only assessed reasons for stopping breakfast and the main meal and did not explore other mealtimes or snacking behaviors, which may be important in understanding overall dietary patterns among adults in the UAE.

## Conclusion

The findings of this study provide valuable insights into the dietary habits and lifestyle factors that may impact food intake among adults in the UAE. The results suggest that there is a need for targeted nutrition interventions to address the specific challenges faced by these participants, including inadequate physical activity, low water intake, and poor consumption of fruits, vegetables, and dairy products. In addition, the findings emphasize the need to incorporate factors linked to physical satisfaction and the planned amount of food when creating nutrition interventions to promote healthy eating habits among adults in the UAE.

## Supporting information

S1 Table The mean score for the five scales of the RISE-Q for breakfast and main meal among study participants. (DOCX)
