# Supplementary material for: Reasons individuals stop eating questionnaire (RISE-Q) among adults in the United Arab Emirates
Source: PLoS One. 2023 Oct 25;18(10):e0293386. doi: 10.1371/journal.pone.0293386 (PMC10599582; doi:10.1371/journal.pone.0293386)
Supplement: S1 Table — (DOCX) [file pone.0293386.s001.docx]

|  | **Breakfast (n=835)** | **Main Meal**  **(n=1482)** |
| --- | --- | --- |
| **Scale** | **Mean ± SD** | **Mean ± SD** |
| Decreased Food Appeal | 8.0 ± 3.9 | 8.2 ± 4.0 |
| Physical Satisfaction | 14.6 ± 3.3 | 14.8 ± 3.6 |
| Planned Amount | 14.9 ± 3.7 | 14.8 ± 3.8 |
| Self-Consciousness | 8.3 ± 3.6 | 8.2 ± 3.7 |
| Decreased Priority of Eating | 8.8 ± 3.4 | 8.8 ± 3.6 |

**S1 Table. The mean score for the five scales of the RISE-Q for breakfast and main meal among study participants**
